# Medical and Physical Intelligence

**Published:** 1802-04

**Authors:** 


### MEDICAL AND PHYSICAL INTELLIGENCE.

[ FOREIGN AND DOMESTIC. ]

---

### To the Editors of the Medical and Physical Journal.

Gentlemen,

As I believe every unprejudiced mind muft, at the prefent moment, be fufficiently convinced of the great utility of the Vaccine Inoculation, permit me, with all due deference to my much honoured brethren in the Profeffion, to propofe, That as a plan of national remuneration to the valued Difcoverer is now before a Committee of the Houfe of Commons, that in order to give that Committee a more ample idea of its efficacy, there fhould be a general confirmation of the fame by the voluntary teftimonies of every friend to the Inoculation throughout the kingdom.

In order to accomplifh this end, I beg leave to propofe, that there be a Committee formed in every city and town throughout Great

Great Britain: That such Committee shall be composed of Medical Gentlemen only, who, laying aside all private differences, shall jointly come forward to support and confirm so honourable a testimony: That such Committee shall draw up a Memorial of their belief of its efficacy, and subscribe the same with their names and places of abode; and that as many sincere friends to the Discovery reside at distances from large towns, notice shall be given to them by the Secretary of the nearest town, intimating to them the nature of the business; and that the Memorial will lay at a certain place for their signatures for a prescribed time: That these Memorials shall be transmitted, as early as possible, to the care of the Editors of the Medical and Physical Journal, (post paid) to be forwarded to Dr. JENNER.

And that while our Brethren in the country are thus laudably engaged, we may follow the example of the Medical Society of Guy's Hospital, by appointing a Committee to receive the signatures of those Gentlemen in London, and its vicinity, who are feelingly alive to the importance of the practice, of rescuing our fellow-creatures from the destructive and mutilating effects of that scourge to the human race, the Small-pox; a disease which, like a mighty torrent, has, for many years past, swept away the best hopes of individuals in particular, and this nation in general. I humbly think, we need no other proof of the superiority of the Vaccine Inoculation than the Bills of Mortality for the preceding year, in which we see the number of deaths from the Small-pox were decreased from the number of the former year full 1000.

I hope I shall be believed when I say that I have not the smallest knowledge of Dr. Jenner, and that my motive in this arises only from a desire to the farther diffusion of so beneficent and important a Discovery, which has now triumphed over so much opposition; and that as I honour the Discovery, I desire, in conjunction with every well-wisher to his country and the community, that an adequate Reward should be given to the Discoverer.

I trust all your Readers will see this subject in its true light; and though some of them, perhaps, may think this Communication useless, I hope they will look upon it as it really is, only as a Hint which I wish may be improved upon by a more able pen than him who begs leave to subscribe himself,

Your's, &c.

*London, March* 19, 1802. J. C KNIGHT.

---

The incalculable loss which this kingdom, and indeed the whole world, has sustained by the death of the Duke of Bedford, will naturally turn the attention of the Public to the important subject of HERNIA. The number of fatal Cases which comes to the knowledge of professional men, is sufficient to keep their attention always awake to the danger of neglecting any kind of Rupture, especially those which permit any considerable portion of intestine

 to to protrude; for though a patient, or his furgeon, may replace the bowel with great eafe a thoufand times, yet, in fome fatal moment, as happened in this much-lamented inftance, a fudden contraction of the orifice may take place, and the reduction be rendered impoffible without an operation. The danger of the operation, even in the moft fkilful hands, is fufficiently known to make us anxious to endeavour to prevent the necessity of it by the ufe of proper Truffes and Bandages.

---

To the EDITORS of the MEDICAL and PHYSICAL JOURNAL.

Gentlemen,

AS we have, at the particular defire of feveral profeffional men of the firft refpectability, avoided all the common modes by which our invention of a Trufs might have been more generally known to the public at large, we have to folicit that it may have a place in your valuable Publication, that we may, through fo refpectable a medium, have the opportunity of making it known to the Faculty in general.

Our Patent was obtained for the *art of bending tempered steel without the use of heat*. Upon fubmitting this difcovery to feveral Surgeons of the firft eminence, they have given it as their individual opinions, after the moft pofitive and repeated proofs of its operation under their immediate infpection, that a Trufs made upon this principle is far fuperior to any ever yet invented, both for *durability and effect*, befides their having this peculiar advantage, that they are worn without ftraps.

We have no doubt but they will be, when known, generally adopted; and we are fanctioned in this opinion by having it in our power to fay, that the Army Elaboratory now ufe them to the exclufion of all others; the Royal Hofpitals of Greenwich and Chelfea, including out-penfioners, invalids, &c. the York Hofpital, &c. entirely ufe them; the St. George's Hofpital, the Mary-le-Bone Infirmary and Workhoufe; the Greenwich alfo, and other Inftitutions, have adopted them. Thefe are facts which will, the Patentees truft, fpeak loud in their behalf; however, it is the merit of the invention, and the judgment of thofe who are competent to decide, that they wifh fhould recommend them to notice.

It may not be improper to mention, that the Patentees, who are gun-makers, No. 37, Charing Crofs, vend them entirely themfelves; nor do they make any fhow of this article.

We are, &c. &c.

*Feb.* 24, 1802. TATHAM AND EGG.

---

Dr. PRIESTLEY has replied to Mr. CRUIKSHANK'S Defence of his new Syftem of Chemiftry. He is ftill the zealous Advocate for the doctrine of phlogifton, and calls upon Mr. Cruikfhank to re-confider his hypothefis, and extend his examination to all the other arguments advanced in favour of the phlogiftic fyftem, and againft the decompofition of water.

---

In

In another communication addreffed to Mr. Nicholson, Dr. Priestley has detailed a number of obfervations and experiments relating to the Pile of Volta, which feem favourable to the hypothefis of two electric fluids; the pofitive containing the principal of oxygen, and the negative, that of phlogifton. Thefe united to water, conftitute the two kinds of air, viz. dephlogifticated and inflammable. He fays they tend likewife to confirm a conjecture advanced by himfelf many years ago, refpecting the fimilarity of the electric matter and phlogifton, and, together with proper Galvanic experiments, fhew that the fame fubftance elaborated from the aliment by the brain is the caufe of mufcular motion, the nerves being the moft fenfible of all electrometers.

---

In Scherer's Chemical Journal, Number 40, we have an analyfis of fome bituminous wood, made by R. Jameson, at Freiberg, who conceives that he has difcovered a new acid, which cannot be cryftallized, and which, by evaporation, is feen under the form of fmall fhells or fcales, of an acidulous tafte. It is difficult of folution when combined with lime; it decompofes all the nitrat and acetat of lead; it produces a brown precipitate in the fulphat of copper; in the fulphat of iron, the colour of the precipitate is of a deeper brown. The folution with the nitrat of copper takes a beautiful green colour, without any precipitation. It decompofes alfo the nitrat and muriat of barytes. This acid, mixed with a folution of indigo, in the fulphurous acid, produces a fine green colour. It refembles carbonic acid the moft, and, when poured on carbon, it forms a brown and bitter matter, which is foluble in water, fpirits of wine, and alkaline folutions. It then gives out a very penetrating and aromatic odour. The author thinks that the acid is compofed only of carbon and oxygen.

---

The Medical Repofitory of New York, contains the following dreadful picture of Irifh Emigration. Several fhips which had carried flax-feed to Ireland returned in the months of June and July to New York, crowded with needy and wretched emigrants from that ifland. They were fo thick between decks that the air was deprived of its ufual portion of oxygen, infomuch, that on bringing the fick paffengers to fhore, the common pure atmofphere was too ftimulant for their lungs, and a number of them gafped in it, and died in a fhort time. There was fo much animal excrement accumulated in one of the fhips, that the Health-officer detained her at the quarantine ground as poifonous and peftilential, and refufed to let her come up to the city. By the pukings and purgings, and by the urinary and perfpiratory difcharges of thefe miferable creatures, literally wallowing in their own filth, the bodies of many of them were befmeared and incrufted, forming a layer of excrementitious grime from head to foot. Their clothing and their bedding were impregnated with as much of thefe excrementitious matters as they could wipe from the bodies of the paffengers and abforb. And with fuch coverings, vile, offenfive, and peftilential in the higheft degree, were they furrounded. And thefe excrements, infefting every thing in the neighbourhood of the fick, underwent the ufual chemical chemical changes in a heat nearly or quite equal to the human body, and turned to feptic acid, or to fome other feptic and poifonous matter, which forms the exciting caufe of fever. Of the fever fo excited, between thirty and forty from one fhip died in croffing the Atlantic, and were thrown overboard. The furvivors arrived in a ftate of uncleanlinefs, ficknefs and want, feldom feen in America but among the emigrants from that unhappy country, who make fo large a number of the poor in the American hofpitals and alms-houfes. So thoroughly contaminated with their own corrupting excretions were the clothes and beds of thefe fufferers, that the feptic exhalations from them poifoned the air of the Marine Hofpital, on Staten Ifland, and the medical attendants and nurfes fickened in the difcharge of their humane attentions. Mr. Bayley, the Health-officer of the port of New York, caufed the fick, after landing, immediately to be diftributed or feparated from each other as widely as the circumftances would allow, that their peftilential exhalations might be diluted, and wafted off. He ordered their nafty clothing and bedding to be carried away from their perfons, and that part of both which was too poifonous, ragged and rotten to be worth the cleaning, to be burned or thrown into the Bay. He directed the bodies of the fick to be purified by careful ablution and fcrubbing with a folution of foap in water; and even the heads of fome of them to be fhaven. After thefe things were done, the fick were furnifhed with clean clothing and bedding from the public ftore. The walls of the hofpital were repeatedly white-wafhed with lime, and the floors and utenfils fcrubbed with alkaline ley of potafh.

So offenfive and intolerable were many of thefe languifhing creatures, that they were accommodated under large tents, for the benefit of more complete airing; and it was remarked, that the ground on which the tents were pitched grew too unfafe, in a few days, to be dwelt upon any longer, and the tents were removed, and erected upon frefh portions of earth. In fuch cafes, the envenomed and deferted fpot was regularly fprinkled over with lime. The Health-Officer, knowing that hard or bar foap was made of foda, and contained withal, as manufactured in New York, a large quantity of turpentine, which only added to its weight without increafing its virtue, procured for the ufe of the wafh-houfe a ftronger foap, made of the more powerful alkali, pot-afh, combined with juft enough of animal fat to leffen its caufticity fo as to bear handling. With this very efficacious foap, and not with the common mixture of turpentine, flufh and foda, in the fhops, were the remaining clothes cleanfed and alkalized. The confequence of this management was, that as foon as thefe regulations could be carried into effect, the peftilential vapours were difperfed through the furrounding atmofphere; a vivifying air was admitted into the lungs; the peftilential matter adhering to the bodies and clothes was alkalized and overcome, and poifonous effluvia iffued from them no longer. It is worthy of being remembered, that Emigrants from Ireland, landing immediately in the city, inftead of being detained at the Marine Hofpital, filled New York with death and terror in in 1795: The Editors of the Medical Repofitory, add to this ftatement the following reflection: "The benevolent and philofophical gentlemen of Ireland would be worthily employed in preventing thefe calamities, if poffible, among their countrymen, and thereby relieving the United States from fuch fhocking fcenes."

---

*Another instance of pestilence engendered in a ship crowded with passengers from Ireland, copied from a subsequent number of the Medical Repository.* The fhip Nancy, Capt. J. Heron, was chartered by a commercial houfe at Sligo, to carry paffengers from that port to New York. She failed from Sligo on the 12th of July, 1801, and arrived, after a paffage of feventy-feven days, at the port of New York, on the 27th of September following. This fhip, of the burthen of 202 tons, received on board 417 paffengers, and was navigated by nine feamen. The provifions, mere refufe, put up by Contractors with the view of faving expence, were of the worft kind; and the water, which was alfo of bad quality from the unexpected length of the voyage, became extremely fcanty before the arrival of the fhip. In order to receive fo great a number of paffengers on board this fhip, temporary cabins were built on the quarter deck, which were filled with eighty perfons, and three hundred were crowded into the fpace between decks. It will excite no furprife that a veffel, thus crowded, became fickly foon after failing from Sligo. Typhus fever and dyfentery foon began to prevail, and deftroyed the lives of a large proportion of the paffengers. In addition to the wretchednefs of being confined in fuch numbers in fo fmall a fpace, thefe unhappy Emigrants fuffered all the evils which their habits of uncleanlinefs could produce. Their bodies and clothes, covered and faturated with filth, exhaled poifon all around them. Partly from the want of ftrength and affiftance among the fick, and partly from the want of a fenfe of decency, the fpace between decks, occupied by nearly 300 perfons, became the receptacle of all excremental matters, infomuch that they iffued in fteams from the fcuppers. The filth on the upper deck was nearly over the fhoes. The fides of the fhip were daubed and incrufted with excrements; and even the ropes for the fupport of fuch as wifhed to go on board, were unfit to be handled: The ftench was intolerably offenfive. In fuch condition arrived this unfortunate veffel at the place affigned for quarantine in the port of New York. Ninety perfons had died on the paffage; one hundred and eighty were fick; fcarcely a healthy countenance was to be feen on board of the fhip; very few had efcaped difeafe, and many had fuffered from three to four relapfes. About forty were taken ill after their arrival. As foon as poffible the fick were brought on fhore, ftripped of their filthy and peftilential clothes, their bodies thoroughly wafhed and fcoured with foap and water, and then wrapped up in clean blankets, and carried into the wards appointed for their reception in the Marine Hofpital. The permanent buildings of the eftablifhment were infufficient to receive fo great a number; tents and other temporary accommodations were provided for the remainder. Separation, ventilation, and cleanlinefs, cleanlinefs, as foon as they could be brought into action, accomplifhed every thing that could be expected; and only twenty-fix have died fince their arrival at this port.

---

Dr. Batty's Lectures on Midwifery and on the Difeafes of Women and Children, will commence at his houfe in Marlborough Street, on Monday, April 12, at eleven o'clock in the morning.

---

Dr. Dennison and Dr. Squire will begin, early in the next month, a Courfe of Lectures on the Theory and Practice of Midwifery, and the Difeafes of Women and Children. Notice of the day will be advertifed in the public papers.

---

We underftand that Mr. Allen will begin his Courfe of Lectures on the Animal Economy at Edinburgh, in the beginning of May. In thefe lectures the phyfiology of the human body is explained, and various pathological facts illuftrated, with all that precifion and accuracy for which this gentleman is fo juftly and fo eminently diftinguifhed. We may remark, that Mr. Allen has the merit of being one of the firft public teachers who introduced into notice thofe philofophical views of this branch of knowledge, which are daily becoming more general, and which promife fo much advantage to the art of medicine, as well as to phyfical fcience.

---

On Wednefday, Feb. 24, Dr. Babington, Affiftant Phyfician to Guy's Hofpital, was unanimoufly appointed by the Governors, Phyfician in Ordinary, in the room of Dr. Saunders, refigned. And on Wednefday the 10th of March, came on the election of an Affiftant Phyfician, when Dr. James Curry, of Old Broad-ftreet, and formerly Phyfician to the Northampton Hofpital, was unanimoufly chofen to that office.

---

Shortly will be publifhed, Strictures upon the Obfervations of Mr. Crowfoot; with an Appendix, containing a Copy of Doctor Moffman's ingenious Paper on Apoplexy; together with Doctor Langflow's Anfwer to Meffrs. Wilkinfon and Atkinfon.

